# *N*-[(1*S*,2*S*)-2-Amino-1,2-diphenyl­eth­yl]-4-methyl­benzene­sulfonamide [(*S*,*S*)-TsDPEN]

**DOI:** 10.1107/S1600536810049159

**Published:** 2010-11-27

**Authors:** Claudine Schlemmer, Dieter Schollmeyer, Nancy Blank, Alexander Stoye, Till Opatz

**Affiliations:** a Institut für Organische Chemie, Universität Mainz, Duesbergweg 10-14, 55128 Mainz, Germany

## Abstract

The crystal structure of the title compound, C_21_H_22_N_2_O_2_S, shows a network of N—H⋯N and N—H⋯O hydrogen bonds. The tolyl and 1-phenyl rings are almost mutually coplanar [7.89 (9)°], while the 2-phenyl ring makes a dihedral angle of 50.8 (1) ° with the 1-phenyl ring. An intra­molecular N—H⋯N hydrogen bond stabilizes the mol­ecular conformation.

## Related literature

For the synthesis of the title compound, see: Vanino (1923 ▶); Mistryukov (2002 ▶). The title compound was synthesized as a ligand for Ru-catalyzed asymmetric transfer hydrogenations. Similar to BINAP introduced by the same author, the synthesized diamine permits highly enanti­oselective asymmetric hydrogenation reactions, see: Noyori (1996 ▶).
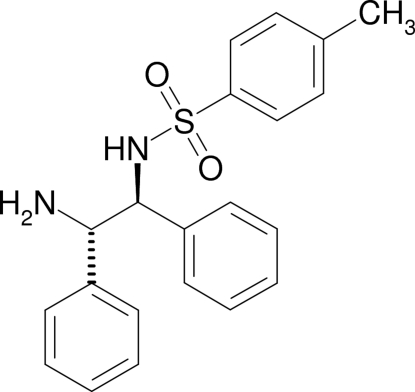


## Experimental

### 

#### Crystal data

C_21_H_22_N_2_O_2_S
*M* *_r_* = 366.47
Orthorhombic, 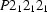

*a* = 6.3892 (6) Å
*b* = 12.2290 (11) Å
*c* = 24.281 (2) Å
*V* = 1897.2 (3) Å^3^
*Z* = 4
Mo *K*α radiation
μ = 0.19 mm^−1^
*T* = 173 K
0.50 × 0.05 × 0.05 mm

#### Data collection

Bruker APEXII CCD diffractometer
37668 measured reflections
4511 independent reflections
3865 reflections with *I* > 2σ(*I*)
*R*_int_ = 0.062

#### Refinement

*R*[*F* ^2^ > 2σ(*F* ^2^)] = 0.036
*wR*(*F* ^2^) = 0.086
*S* = 1.02
4511 reflections
236 parameters
H-atom parameters constrained
Δρ_max_ = 0.30 e Å^−3^
Δρ_min_ = −0.30 e Å^−3^
Absolute structure: Flack (1983 ▶), 1905 Friedel pairs
Flack parameter: 0.01 (7)

### 

Data collection: *APEX2* (Bruker, 2006 ▶); cell refinement: *SAINT* (Bruker, 2006 ▶); data reduction: *SAINT*; program(s) used to solve structure: *SIR97* (Altomare *et al.*, 1999 ▶); program(s) used to refine structure: *SHELXL97* (Sheldrick, 2008 ▶); molecular graphics: *PLATON* (Spek, 2009 ▶); software used to prepare material for publication: *PLATON*.

## Supplementary Material

Crystal structure: contains datablocks I, global. DOI: 10.1107/S1600536810049159/bt5420sup1.cif

Structure factors: contains datablocks I. DOI: 10.1107/S1600536810049159/bt5420Isup2.hkl

Additional supplementary materials:  crystallographic information; 3D view; checkCIF report

## Figures and Tables

**Table 1:** Hydrogen-bond geometry (Å, °)

*D*—H⋯*A*	*D*—H	H⋯*A*	*D*⋯*A*	*D*—H⋯*A*
N11—H11⋯N20^i^	0.97	2.09	3.041 (2)	167
N20—H20*A*⋯N11	0.99	2.31	2.8149 (19)	110
N20—H20*B*⋯O10^i^	0.87	2.17	3.0236 (19)	166

Symmetry code: (i) 
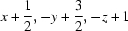
.

**Fig. 1.**
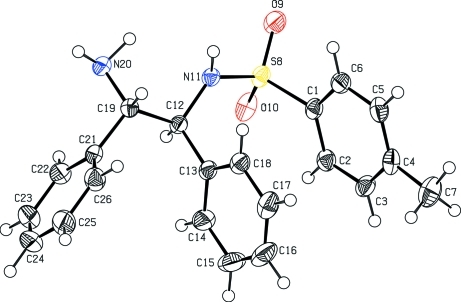
View of compound I. Displacement ellipsoids are drawn at the 50% probability level.

**Fig. 2.**
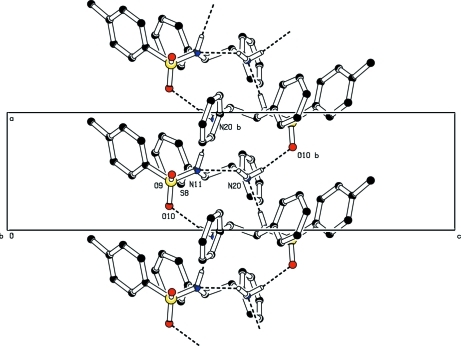
Part of the packing of I showing the hydrogen bond network. View along b axis.

## supplementary crystallographic information

### Comment

The title compound is formed from hydrobenzamide in a base catalyzed one-pot reaction involving deprotonation to a diazapentadienide anion, cyclization to amarine, and isomerization at elevated temperature under formation of isoamarine. Subsequent reduction with Al/Hg then furnishes racemic stilbenediamine. To obtain optically pure *(S,S)—N-p*-toluenesulfonyl-1,2-diphenylethylenediamine, the racemate is converted to the diastereomeric *L*-tartrate salts which are separated by crystallization. After conversion to the free base, the *(S,S)*-1,2-diphenylethylenediamine is reacted with *p*-toluenesulfonyl chloride to give the desired product as colorless crystals. The crystal structure is characterized by a network consisting of the three following hydrogen bonds: N11—H11···N20 (2.09 Å), N20—H20B···O10 (2.17 Å) and N20—H20A···N11 (2.31 Å). The first two bonds build the network to a second molecule while the last one stabilizes the molecular conformation. Furthermore, the aromatic rings C1—C6 and C13—C18 are almost coplanar while the ring C21–26 exhibits a dihedral angle of 50.8 (1) ° to the ring C13—C18. The carbon atoms C12 and C19 are (S)-configurated.

### Experimental

The title compound was prepared from hydrobenzamide (Vanino, 1923) as follows: To a suspension of hydrobenzamide (66 g, 0.22 mol) in dry DMSO (100 ml) was added solid NaOH (1.2 g, 0.03 mol) under argon atmosphere and vigorous stirring. After 5 min, the mixture was heated to 323 K and held at this temperature for 1 h. Then, the reaction mixture was heated to 403 K and stirred at that temperature for another 3 h. The mixture was cooled down to 353 K and diluted with ethanol and ammonia (28% in water). For completion of the crystallization, the mixture was allowed to stand at room temperature for 20 h. The crystalline isoamarine was filtered off and washed with 2-propanol (Mistryukov, 2002). Yield: 57.2 g (87%, Lit.:90%), m.p.: 474 K (Lit.: 471–473 K). In an argon atmosphere a mixture of isoamarine (50 g, 0.17 mol), pieces of aluminium foil (13.5 g, 0.5 mol), and HgCl_2_ (3 g, 0.011 mol) were suspended in dry THF (300 ml). The suspension was stirred for 15 min. Then, water (9 ml) in THF (25 ml) was added during 1.5 h. After 2 h, another portion of water (25 ml) was added and the mixture was allowed to stand for 20 h. A concentrated solution of aqueous ammonia (50 ml) was added and the mixture was set aside for another 24 h before it was filtered. The filtrate was concentrated and the distillate was used to wash the residue on the filter. The obtained filtrate was also concentrated and combined with the remainder obtained in the first evaporation. The resulting oil was dissolved in a mixture of methanol (150 ml), concentrated HCl (75 ml) and water (50 ml). The crystaline dihydrochloride was filtered off, washed with dioxane and dried. To obtain the free base, the salt was dissolved in water (100 ml), NaOH was added and the solution was extracted four times with CHCl_3_. The combined organic layers were concentrated to dryness (see Mistryukov (2002)). Yield: 19.0 g (53%, Lit.: 78%), m.p.: 354 K (Lit.: 355 K). The obtained diamine (14.5 g, 0.068 mol) was dissolved in ethanol (77 ml) and warmed to 343 K. To this solution, *L*-(+)-tartaric acid (10.2 g, 0.068 mol) dissolved in hot (343 K) ethanol (77 ml) was added. The mixture was allowed to cool to ambient temperature before filtration. The crystals were washed with ethanol and dried *in vacuo*. The salt was then dissolved in boiling water (77 ml), ethanol (77 ml) was added and the mixture was allowed to cool slowly to room temperature. This procedure was repeated twice to give the desired *(S,S)*-diamine-*(R,R)*-tartrate salt. Yield: 6.0 g (48%, Lit.: 63–69%), m.p.: 465 K, [α]_D_^23^: -10.4° (H_2_O, c=1.0), Lit.: -10.8° (H_2_O, c=1.3). The salt was suspended in water (80 ml), cooled to 273 K and 8 ml of 50% aqueous solution of NaOH were added dropwise. The solution was extracted with CH_2_Cl_2_, the combined organic layers were washed with brine and dried over anhydrous Na_2_SO_4_. The solvent was evaporated *in vacuo*. From the combined filtrates and mother liquors, the *(R,R)*-diamine can be obtained in a similar fashion by crystallization with D-(–)-tartaric acid. Yield: 3.2 g (44%, Lit.: 57–66%), m.p.: 356 K, Lit.: 356 K, [α]_D_^23^: -101.9° (MeOH, c=1.0), Lit.: -106.0° (MeOH, c=1.1). For the tosylation, *(S,S)*-1,2-diphenylethylenediamine (0.3 g, 1.41 mmol) was dissolved in dry CH_2_Cl_2_ (3 ml) and Et_3_N (320 µL) was added. Then, *p*-toluenesulfonyl chloride (0.256 g, 1.41 mmol) dissolved in dry CH_2_Cl_2_ (9 mL) was added dropwise. After 30 min of stirring, the solution was diluted with CH_2_Cl_2_ to twice its volume and washed with saturated NaHCO_3_, brine and dried over anhydrous Na_2_SO_4_. After evaporation of the solvent, the product was purified by flash chromatography on silica (petroleum ether/ethyl acetate, 2:3) and subsequently recrystallized from benzene. Yield: 0.33 g (62%, Lit.: 49%), m.p.: 403 K, [α]_D_^23^: -90° (MeOH, c=1.0), Lit.: -89° (MeOH, c=1.0).

### Refinement

Hydrogen atoms attached to carbons were placed at calculated positions with C—H = 0.95 Å (aromatic) or 0.98–0.99 Å (*sp*^3^ C-atom). Hydrogen atoms attached to nitrogen were located in difference Fourier maps. All H atoms were refined in the riding-model approximation with isotropic displacement parameters (set at 1.2–1.5 times of the *U*_eq_ of the parent atom).

### Crystal data

**Table:**

C_21_H_22_N_2_O_2_S	*D*_x_ = 1.283 Mg m^−^^3^
*M**_r_* = 366.47	Melting point: 403 K
Orthorhombic, *P*2_1_2_1_2_1_	Mo *K*α radiation, λ = 0.71073 Å
Hall symbol: P 2ac 2ab	Cell parameters from 6316 reflections
*a* = 6.3892 (6) Å	θ = 2.3–26.7°
*b* = 12.2290 (11) Å	µ = 0.19 mm^−^^1^
*c* = 24.281 (2) Å	*T* = 173 K
*V* = 1897.2 (3) Å^3^	Needle, colourless
*Z* = 4	0.50 × 0.05 × 0.05 mm
*F*(000) = 776	

### Data collection

**Table:**

Bruker APEXII CCD diffractometer	3865 reflections with *I* > 2σ(*I*)
Radiation source: sealed Tube	*R*_int_ = 0.062
graphite	θ_max_ = 27.9°, θ_min_ = 1.9°
CCD scan	*h* = −8→8
37668 measured reflections	*k* = −16→16
4511 independent reflections	*l* = −31→31

### Refinement

**Table:**

Refinement on *F*^2^	Secondary atom site location: difference Fourier map
Least-squares matrix: full	Hydrogen site location: inferred from neighbouring sites
*R*[*F*^2^ > 2σ(*F*^2^)] = 0.036	H-atom parameters constrained
*wR*(*F*^2^) = 0.086	*w* = 1/[σ^2^(*F*_o_^2^) + (0.0404*P*)^2^ + 0.3917*P*] where *P* = (*F*_o_^2^ + 2*F*_c_^2^)/3
*S* = 1.02	(Δ/σ)_max_ < 0.001
4511 reflections	Δρ_max_ = 0.30 e Å^−^^3^
236 parameters	Δρ_min_ = −0.30 e Å^−^^3^
0 restraints	Absolute structure: Flack (1983), 1905 Friedel pairs
Primary atom site location: structure-invariant direct methods	Flack parameter: 0.01 (7)

### Special details

**Table:**

Geometry. All e.s.d.'s (except the e.s.d. in the dihedral angle between two l.s. planes) are estimated using the full covariance matrix. The cell e.s.d.'s are taken into account individually in the estimation of e.s.d.'s in distances, angles and torsion angles; correlations between e.s.d.'s in cell parameters are only used when they are defined by crystal symmetry. An approximate (isotropic) treatment of cell e.s.d.'s is used for estimating e.s.d.'s involving l.s. planes.
Refinement. Refinement of *F*^2^ against ALL reflections. The weighted *R*-factor *wR* and goodness of fit *S* are based on *F*^2^, conventional *R*-factors *R* are based on *F*, with *F* set to zero for negative *F*^2^. The threshold expression of *F*^2^ > σ(*F*^2^) is used only for calculating *R*-factors(gt) *etc*. and is not relevant to the choice of reflections for refinement. *R*-factors based on *F*^2^ are statistically about twice as large as those based on *F*, and *R*- factors based on ALL data will be even larger.

### Fractional atomic coordinates and isotropic or equivalent isotropic displacement parameters (Å^2^)

**Table:**

	*x*	*y*	*z*	*U*_iso_*/*U*_eq_	
C1	0.5561 (3)	0.73082 (14)	0.30984 (7)	0.0254 (4)	
C2	0.4667 (3)	0.64651 (15)	0.27929 (7)	0.0279 (4)	
H2	0.3271	0.6237	0.2863	0.033*	
C3	0.5848 (3)	0.59602 (15)	0.23830 (7)	0.0306 (4)	
H3	0.5234	0.5399	0.2166	0.037*	
C4	0.7904 (3)	0.62644 (18)	0.22876 (7)	0.0329 (5)	
C5	0.8743 (3)	0.71278 (18)	0.25899 (7)	0.0335 (4)	
H5	1.0134	0.7361	0.2516	0.040*	
C6	0.7597 (3)	0.76556 (16)	0.29955 (7)	0.0296 (4)	
H6	0.8189	0.8242	0.3199	0.036*	
C7	0.9227 (4)	0.5637 (2)	0.18787 (9)	0.0502 (6)	
H7A	0.9553	0.4913	0.2029	0.075*	
H7B	1.0530	0.6037	0.1810	0.075*	
H7C	0.8454	0.5555	0.1533	0.075*	
S8	0.42061 (7)	0.78898 (4)	0.366248 (17)	0.02678 (11)	
O9	0.4722 (2)	0.90309 (10)	0.36804 (5)	0.0385 (4)	
O10	0.2061 (2)	0.75474 (12)	0.36209 (5)	0.0361 (3)	
N11	0.5121 (2)	0.73717 (11)	0.42319 (6)	0.0236 (3)	
H11	0.6453	0.7683	0.4344	0.028*	
C12	0.4568 (3)	0.62583 (14)	0.44193 (7)	0.0223 (4)	
H12	0.3007	0.6214	0.4434	0.027*	
C13	0.5326 (3)	0.53456 (15)	0.40392 (7)	0.0247 (4)	
C14	0.3947 (4)	0.45222 (16)	0.38829 (8)	0.0366 (5)	
H14	0.2564	0.4518	0.4026	0.044*	
C15	0.4569 (4)	0.37034 (18)	0.35183 (9)	0.0466 (6)	
H15	0.3614	0.3144	0.3415	0.056*	
C16	0.6570 (4)	0.37089 (19)	0.33092 (9)	0.0466 (6)	
H16	0.6993	0.3159	0.3056	0.056*	
C17	0.7967 (4)	0.45140 (18)	0.34668 (8)	0.0393 (5)	
H17	0.9351	0.4512	0.3324	0.047*	
C18	0.7356 (3)	0.53278 (15)	0.38341 (7)	0.0291 (4)	
H18	0.8330	0.5872	0.3945	0.035*	
C19	0.5390 (3)	0.61293 (14)	0.50177 (7)	0.0226 (4)	
H19	0.6944	0.6224	0.5015	0.027*	
N20	0.4464 (2)	0.70033 (12)	0.53631 (6)	0.0268 (3)	
H20A	0.5038	0.7658	0.5174	0.032*	
H20B	0.5100	0.7034	0.5679	0.032*	
C21	0.4890 (3)	0.50046 (15)	0.52464 (7)	0.0257 (4)	
C22	0.2902 (3)	0.47483 (17)	0.54406 (8)	0.0328 (4)	
H22	0.1833	0.5288	0.5440	0.039*	
C23	0.2476 (4)	0.36990 (19)	0.56357 (9)	0.0434 (5)	
H23	0.1118	0.3528	0.5770	0.052*	
C24	0.4014 (4)	0.29086 (18)	0.56347 (9)	0.0469 (6)	
H24	0.3706	0.2193	0.5763	0.056*	
C25	0.5992 (4)	0.31529 (17)	0.54489 (9)	0.0456 (6)	
H25	0.7054	0.2609	0.5451	0.055*	
C26	0.6430 (3)	0.41983 (16)	0.52574 (8)	0.0343 (5)	
H26	0.7801	0.4366	0.5132	0.041*	

### Atomic displacement parameters (Å^2^)

**Table:**

	*U*^11^	*U*^22^	*U*^33^	*U*^12^	*U*^13^	*U*^23^
C1	0.0288 (10)	0.0289 (9)	0.0185 (7)	0.0034 (8)	−0.0029 (7)	0.0021 (7)
C2	0.0279 (10)	0.0324 (10)	0.0232 (8)	−0.0010 (8)	−0.0036 (7)	0.0037 (7)
C3	0.0362 (11)	0.0336 (10)	0.0219 (8)	0.0003 (10)	−0.0040 (8)	−0.0023 (7)
C4	0.0356 (12)	0.0424 (12)	0.0208 (9)	0.0060 (10)	0.0021 (8)	0.0058 (8)
C5	0.0282 (11)	0.0455 (11)	0.0268 (9)	−0.0045 (10)	0.0025 (7)	0.0062 (9)
C6	0.0316 (10)	0.0318 (11)	0.0253 (9)	−0.0034 (8)	−0.0040 (8)	0.0013 (7)
C7	0.0485 (14)	0.0662 (16)	0.0360 (11)	0.0062 (13)	0.0115 (11)	−0.0073 (11)
S8	0.0311 (2)	0.0291 (2)	0.02014 (19)	0.0075 (2)	−0.00348 (19)	0.00053 (18)
O9	0.0594 (10)	0.0284 (7)	0.0276 (6)	0.0100 (6)	−0.0056 (7)	0.0018 (6)
O10	0.0263 (7)	0.0561 (9)	0.0258 (6)	0.0114 (6)	−0.0016 (6)	−0.0028 (6)
N11	0.0281 (8)	0.0228 (7)	0.0197 (7)	0.0006 (6)	−0.0035 (6)	0.0000 (6)
C12	0.0243 (9)	0.0228 (8)	0.0199 (7)	0.0010 (7)	−0.0007 (7)	−0.0009 (6)
C13	0.0314 (11)	0.0234 (9)	0.0193 (8)	0.0027 (8)	−0.0039 (7)	0.0007 (7)
C14	0.0457 (13)	0.0328 (10)	0.0314 (10)	−0.0052 (10)	−0.0062 (9)	−0.0017 (8)
C15	0.0673 (17)	0.0312 (11)	0.0413 (12)	−0.0040 (11)	−0.0149 (11)	−0.0086 (9)
C16	0.0717 (18)	0.0353 (12)	0.0328 (11)	0.0188 (12)	−0.0079 (11)	−0.0115 (9)
C17	0.0473 (14)	0.0425 (13)	0.0281 (9)	0.0182 (10)	−0.0010 (10)	0.0016 (9)
C18	0.0349 (11)	0.0278 (10)	0.0245 (8)	0.0061 (9)	−0.0038 (8)	0.0017 (8)
C19	0.0212 (9)	0.0245 (8)	0.0220 (8)	−0.0002 (7)	−0.0007 (7)	0.0004 (7)
N20	0.0325 (9)	0.0267 (7)	0.0213 (6)	−0.0012 (7)	−0.0019 (6)	−0.0015 (6)
C21	0.0321 (10)	0.0267 (9)	0.0184 (8)	−0.0019 (8)	−0.0022 (7)	−0.0002 (7)
C22	0.0351 (12)	0.0326 (11)	0.0306 (10)	−0.0030 (9)	−0.0016 (9)	0.0002 (8)
C23	0.0522 (14)	0.0400 (13)	0.0379 (11)	−0.0142 (12)	0.0048 (10)	0.0047 (10)
C24	0.0753 (17)	0.0275 (10)	0.0380 (11)	−0.0099 (13)	0.0001 (11)	0.0067 (9)
C25	0.0679 (17)	0.0308 (11)	0.0380 (11)	0.0121 (11)	0.0027 (11)	0.0069 (9)
C26	0.0389 (12)	0.0322 (10)	0.0318 (10)	0.0053 (9)	0.0032 (8)	0.0039 (9)

### Geometric parameters (Å, °)

**Table:**

C1—C6	1.391 (3)	C14—C15	1.394 (3)
C1—C2	1.393 (2)	C14—H14	0.9500
C1—S8	1.7694 (18)	C15—C16	1.376 (4)
C2—C3	1.393 (3)	C15—H15	0.9500
C2—H2	0.9500	C16—C17	1.383 (3)
C3—C4	1.385 (3)	C16—H16	0.9500
C3—H3	0.9500	C17—C18	1.392 (3)
C4—C5	1.393 (3)	C17—H17	0.9500
C4—C7	1.512 (3)	C18—H18	0.9500
C5—C6	1.387 (3)	C19—N20	1.482 (2)
C5—H5	0.9500	C19—C21	1.517 (2)
C6—H6	0.9500	C19—H19	1.0000
C7—H7A	0.9800	N20—H20A	0.9933
C7—H7B	0.9800	N20—H20B	0.8686
C7—H7C	0.9800	C21—C22	1.391 (3)
S8—O9	1.4345 (14)	C21—C26	1.393 (3)
S8—O10	1.4367 (14)	C22—C23	1.395 (3)
S8—N11	1.6294 (14)	C22—H22	0.9500
N11—C12	1.478 (2)	C23—C24	1.378 (3)
N11—H11	0.9714	C23—H23	0.9500
C12—C13	1.527 (2)	C24—C25	1.375 (4)
C12—C19	1.553 (2)	C24—H24	0.9500
C12—H12	1.0000	C25—C26	1.389 (3)
C13—C18	1.390 (3)	C25—H25	0.9500
C13—C14	1.391 (3)	C26—H26	0.9500
			
C6—C1—C2	120.92 (17)	C13—C14—C15	120.9 (2)
C6—C1—S8	118.28 (14)	C13—C14—H14	119.6
C2—C1—S8	120.62 (15)	C15—C14—H14	119.6
C1—C2—C3	119.12 (18)	C16—C15—C14	119.7 (2)
C1—C2—H2	120.4	C16—C15—H15	120.2
C3—C2—H2	120.4	C14—C15—H15	120.2
C4—C3—C2	120.95 (19)	C15—C16—C17	120.1 (2)
C4—C3—H3	119.5	C15—C16—H16	120.0
C2—C3—H3	119.5	C17—C16—H16	120.0
C3—C4—C5	118.72 (18)	C16—C17—C18	120.3 (2)
C3—C4—C7	120.2 (2)	C16—C17—H17	119.8
C5—C4—C7	121.0 (2)	C18—C17—H17	119.8
C6—C5—C4	121.56 (18)	C13—C18—C17	120.2 (2)
C6—C5—H5	119.2	C13—C18—H18	119.9
C4—C5—H5	119.2	C17—C18—H18	119.9
C5—C6—C1	118.66 (18)	N20—C19—C21	111.26 (14)
C5—C6—H6	120.7	N20—C19—C12	108.75 (13)
C1—C6—H6	120.7	C21—C19—C12	111.30 (14)
C4—C7—H7A	109.5	N20—C19—H19	108.5
C4—C7—H7B	109.5	C21—C19—H19	108.5
H7A—C7—H7B	109.5	C12—C19—H19	108.5
C4—C7—H7C	109.5	C19—N20—H20A	99.9
H7A—C7—H7C	109.5	C19—N20—H20B	110.1
H7B—C7—H7C	109.5	H20A—N20—H20B	101.6
O9—S8—O10	120.33 (9)	C22—C21—C26	118.62 (18)
O9—S8—N11	105.67 (8)	C22—C21—C19	121.39 (17)
O10—S8—N11	106.77 (8)	C26—C21—C19	119.99 (17)
O9—S8—C1	107.58 (9)	C21—C22—C23	120.1 (2)
O10—S8—C1	107.16 (8)	C21—C22—H22	120.0
N11—S8—C1	108.97 (8)	C23—C22—H22	120.0
C12—N11—S8	122.26 (12)	C24—C23—C22	120.4 (2)
C12—N11—H11	119.0	C24—C23—H23	119.8
S8—N11—H11	113.5	C22—C23—H23	119.8
N11—C12—C13	114.30 (14)	C25—C24—C23	120.2 (2)
N11—C12—C19	107.51 (13)	C25—C24—H24	119.9
C13—C12—C19	112.59 (14)	C23—C24—H24	119.9
N11—C12—H12	107.4	C24—C25—C26	119.7 (2)
C13—C12—H12	107.4	C24—C25—H25	120.2
C19—C12—H12	107.4	C26—C25—H25	120.2
C18—C13—C14	118.82 (18)	C25—C26—C21	121.0 (2)
C18—C13—C12	121.59 (17)	C25—C26—H26	119.5
C14—C13—C12	119.57 (18)	C21—C26—H26	119.5
			
C6—C1—C2—C3	0.7 (3)	C18—C13—C14—C15	−1.3 (3)
S8—C1—C2—C3	−174.34 (14)	C12—C13—C14—C15	177.33 (18)
C1—C2—C3—C4	1.8 (3)	C13—C14—C15—C16	−0.1 (3)
C2—C3—C4—C5	−3.4 (3)	C14—C15—C16—C17	1.0 (3)
C2—C3—C4—C7	174.21 (19)	C15—C16—C17—C18	−0.5 (3)
C3—C4—C5—C6	2.5 (3)	C14—C13—C18—C17	1.8 (3)
C7—C4—C5—C6	−175.05 (19)	C12—C13—C18—C17	−176.82 (16)
C4—C5—C6—C1	−0.1 (3)	C16—C17—C18—C13	−0.9 (3)
C2—C1—C6—C5	−1.5 (3)	N11—C12—C19—N20	57.03 (17)
S8—C1—C6—C5	173.62 (14)	C13—C12—C19—N20	−176.20 (15)
C6—C1—S8—O9	39.30 (17)	N11—C12—C19—C21	179.94 (14)
C2—C1—S8—O9	−145.58 (14)	C13—C12—C19—C21	−53.3 (2)
C6—C1—S8—O10	170.01 (14)	N20—C19—C21—C22	42.9 (2)
C2—C1—S8—O10	−14.86 (17)	C12—C19—C21—C22	−78.6 (2)
C6—C1—S8—N11	−74.81 (16)	N20—C19—C21—C26	−138.06 (17)
C2—C1—S8—N11	100.31 (15)	C12—C19—C21—C26	100.49 (19)
O9—S8—N11—C12	168.30 (14)	C26—C21—C22—C23	−0.7 (3)
O10—S8—N11—C12	39.08 (15)	C19—C21—C22—C23	178.40 (18)
C1—S8—N11—C12	−76.35 (15)	C21—C22—C23—C24	−0.3 (3)
S8—N11—C12—C13	64.27 (19)	C22—C23—C24—C25	1.0 (3)
S8—N11—C12—C19	−169.97 (12)	C23—C24—C25—C26	−0.5 (3)
N11—C12—C13—C18	46.5 (2)	C24—C25—C26—C21	−0.5 (3)
C19—C12—C13—C18	−76.5 (2)	C22—C21—C26—C25	1.1 (3)
N11—C12—C13—C14	−132.06 (17)	C19—C21—C26—C25	−177.99 (18)
C19—C12—C13—C14	104.89 (19)		

### Hydrogen-bond geometry (Å, °)

**Table:**

*D*—H···*A*	*D*—H	H···*A*	*D*···*A*	*D*—H···*A*
N11—H11···N20^i^	0.97	2.09	3.041 (2)	167
N20—H20A···N11	0.99	2.31	2.8149 (19)	110
N20—H20B···O10^i^	0.87	2.17	3.0236 (19)	166

Symmetry codes: (i) *x*+1/2, −*y*+3/2, −*z*+1.
